# Evaluación de las características fisicoquímicas y de la actividad antimicrobiana del aceite del árbol de té contra *Cutibacterium acnes (Propionibacterium acnes)* ATCC 6919

**DOI:** 10.7705/biomedica.5122

**Published:** 2020-12-11

**Authors:** Johana Carolina Ossa-Tabares, Claudia Jimena Llanos, Ana María García

**Affiliations:** 1 Facultad de Ciencias Farmacéuticas y Alimentarias, Departamento de Farmacia, Universidad de Antioquia, Medellín, Colombia Universidad de Antioquia Facultad de Ciencias Farmacéuticas y Alimentarias Departamento de Farmacia Universidad de Antioquia Medellín Colombia; 2 Unidad de Biología Celular y Molecular, Corporación para Investigaciones Biológicas, Medellín, Colombia Corporación para Investiga. Biológicas Corporación para Investigaciones Biológicas Medellín Colombia; 3 Dirección Médico-Científica, Tecnoquímicas, S.A., Medellín, Colombia Tecnoquímicas, S.A Medellín Colombia

**Keywords:** aceite del árbol de té, Propionibacterium acnes, pruebas de sensibilidad microbiana, cromatografía de gases, Tea tree oil, Cutibacterium acnes (Propionibacterium acnes), microbial sensitivity tests, chromatography, gas

## Abstract

**Introducción.:**

El aceite del árbol de té es un aceite esencial reconocido por sus propiedades antimicrobianas.

**Objetivos.:**

Evaluar la composición, características y efecto antimicrobiano del aceite al 2 % del árbol de té y su concentración inhibitoria mínima (CIM) contra *Cutibacterium acnes (Propionibacterium acnes).*

**Materiales y métodos.:**

Se evaluó el quimiotipo en tres lotes diferentes de este aceite mediante cromatografía de gases, así como su actividad antimicrobiana en concentración al 2 % v/v y la CIM contra *C. acnes* mediante ensayo de difusión en agar (guía M11-A8 CLSI).

**Resultados.:**

Los lotes evaluados presentaron los quimiotipos ajustados a la norma ISO 4730, lo que indicó la alta calidad del producto. Los lotes contenían de 30 a 40 % de terpinen-4-ol, compuesto que favorece la actividad antimicrobiana, la cual presentó en todos los lotes un efecto dependiente de la concentración contra *C. acnes,* con una inhibición del crecimiento microbiano en concentración al 2 % v/v en todas las pruebas. La concentración inhibitoria mínima fue de 0,25 % v/v. La actividad antimicrobiana del aceite del árbol de té contra este microorganismo ya ha sido reportada con una concentración inhibitoria mínima entre 0,05 y 1,25 % v/v, rango que cobija la obtenida en este estudio.

**Conclusiones.:**

Los resultados evidenciaron la gran calidad de este producto y su capacidad como agente antibacteriano contra *C. acnes.* Se deben hacer estudios con otros aislamientos del microorganismo provenientes de pacientes con acné vulgar para confirmar su actividad general y la de cada uno de sus componentes.

## Abstract (en)

**Introduction:**

Tea tree oil is an essential oil recognized for its antimicrobial properties.

**Objective:**

To evaluate the composition, features, and antimicrobial effect at 2% v/v, and its minimal inhibitory concentration (MIC) against *Cutibacterium acnes (Propionibacterium acnes).*

**Materials and methods:**

Three different batches of tea tree oil were evaluated. We characterized its chemotype by gas chromatography and its 2% v/v antimicrobial activity against *C. acnes* by agar diffusion assay (guide M11-A8 CLSI).

**Results:**

The three batches of oil had the chemotypes required by the ISO 4730 standard, which indicates that it is a high-quality product. Additionally, they had 30% to 40% of terpinen-4-ol, a compound that favors its antimicrobial activity. Antimicrobial activity against *C. acnes* for all batches had a concentration-dependent effect with microbial growth inhibitory activity in all assays at 2% v/v. The MIC obtained against *C. acnes* for all batches was 0.25% v/v. The antimicrobial activity of tea tree oil against this microorganism has been previously reported with a MIC between 0.05% and 1.25% v/v, a range that covers the one obtained in this study.

**Conclusion:**

These results show the high quality of the oil and its capacity as an antibacterial agent against *C. acnes.* New studies should be conducted to confirm its activity and that of its components in isolates of the microorganism from patients with acne vulgaris.

El aceite del árbol de té es un aceite esencial que se obtiene por destilación de las hojas de gran variedad de plantas del género *Melaleuca,* especialmente de la especie *Melaleuca alternifolia,* planta nativa de Australia. Su potencial terapéutico ha sido reconocido y se destacan sus propiedades antinflamatorias y antimicrobianas ^1^^-^^4^. Debido a su gran producción y comercialización, la guía 4730 de la Organización Internacional para la Estandarización *(International Organization for Standardization,* ISO 4730) indica que la composición del aceite esencial disponible para la venta debe contener una concentración de terpinen-4-ol de 30 % o más y una concentración de 1,8 de cineole de 15 % o menos, además de establecer un rango máximo y mínimo para otros de sus componentes ^5^^,^^6^.

En diversos estudios se describe el amplio espectro antimicrobiano del aceite del árbol de té y de sus componentes, y se ha reportado su efectividad contra un gran número de microorganismos de importancia clínica, como *Escherichia coli, Pseudomonas aeruginosa* y *Staphylococcus* negativo para coagulasa e, incluso, contra cepas de este género resistentes a la meticilina (MRSA) ^1^^,^^4^^,^^7^^-^^9^, bacterias anaerobias Gram positivas y Gram negativas, incluida *Propionibacterium acnes*^2^^,^^3^^,^^10^^,^^11^, virus como el herpes simple ^9^^,^^12^, y hongos de los géneros *Candida, Aspergillus, Penicilliun* y *Trichophyton*^7^^,^^8^^,^^9^^,^^13^. Asimismo, se ha demostrado que el efecto antibacteriano del aceite del árbol de té contra las bacterias anaerobias es más potente que en las aerobias ^4^^,^^14^.

El acné es una enfermedad de la piel causada por la combinación de varios factores de riesgo, incluida la producción excesiva de sebo, la hiperqueratosis y el bloqueo de los poros de la piel, así como la respuestainmunológica e inflamatoria por el crecimiento excesivo de microorganismos; *Cutibacterium acnes (Propionibacterium acnes) es* su principal agente etiológico y desempeña un papel importante en la patogenia de la enfermedad al inducir la producción de mediadores inflamatorios y comedogénicos ^1^^,^^14^^,^^15^^,^^16^. El aceite del árbol de té erradica esta bacteria gracias a su poder antibacteriano ^2^^,^^3^^,^^10^^,^^11^.

El tratamiento para el acné incluye tratamientos tópicos con retinoides o antibióticos y sistémicos con antibióticos orales que, además de causar resistencia de los microorganismos, tienen varios efectos secundarios como irritación cutánea, fotosensibilidad, irritación gastrointestinal y nefrotoxicidad ^14^. Por esto, en estudios recientes se han centrado los esfuerzos en buscar productos de origen natural poco irritantes y efectivos contra este padecimiento ^15^.

Actualmente, hay varios productos cosméticos a base del aceite del árbol de té que se utilizan para el tratamiento del acné, por su poder antibacteriano, su actividad específica contra *C. acnes* y sus propiedades antiinflamatorias ^1^^,^^16^. La importación de este ingrediente activo se hace, sobre todo, de países asiáticos que ofrecen su venta al por mayor. Si bien en muchos casos dichos proveedores suministran información sobre la composición y los límites permitidos para los componentes activos, no proporcionan evidencia de su actividad antimicrobiana general ni de su actividad específica contra *C. acnes.*

En este contexto, el objetivo del estudio fue evaluar las características fisicoquímicas de tres lotes de aceite del árbol de té y su actividad antimicrobiana contra la cepa de *C. acnes* ATCC 6919, determinando su efecto en concentración al 2 % v/v y su concentración inhibitoria mínima (CIM).

## Materiales y métodos

### Producto

Se evaluó la actividad antibacteriana de tres lotes diferentes de aceite del árbol de té producidos por Provital Group (cuadro 1) mediante la destilación por arrastre de vapor de las hojas y la corteza de *Melaleuca alternifolia*^17^.

**Cuadro 1:** Información del fabricante de los lotes de aceite del árbol de té evaluados

Característica	Lote 1 1506107	Lote 2 1510251	Lote 3 1508336
Fecha de producción (D/M/A)	09/02/2015	17/09/2015	25/06/2015
Nombre científico	*Melaleuca alternifolia*	*Melaleuca alternifolia*	*Melaleuca alternifolia*
Producción	Destilación de hojas del árbol del té	Destilación de hojas del árbol del té	Destilación de hojas del árbol del té
Origen	Australia y Polinesia Francesa	Australia y Polinesia Francesa	Australia y Polinesia Francesa

*Evaluación de las características fisicoquímicas y la composición del aceite del árbol de té.* Las características físicas incluyeron el color, la densidad y el índice de refracción. La composición de los tres lotes se evaluó mediante cromatografía de gases acoplada a un detector de ionización de llama, con el fin de analizar el perfil cromatográfico y cuantificar los compuestos del tipo terpinen-4-ol del aceite de *Melaleuca* y de otros presentes en las muestras. Las condiciones de la cromatografía se describen en el cuadro 2.

**Cuadro 2:** Condiciones cromatográficas

Columna	Capilar de sílica fundida
Longitud	50 m
Diámetro interno	0,20 mm
Fase estacionaria	Poli (dimetil siloxano) (OV-101)
Grosor	0,25 µm
Temperatura del horno	Programa de temperatura desde 70 °C a 220 °C con rampa de 2 °C/minuto
Temperatura del inyector	230 °C
Temperatura del detector	250 °C
Detector	Tipo FID
Gas portador	Helio
Volumen de inyección	0,2 µl
Flujo del gas portador	1,0 ml/minuto
Relación de separación	1/100

El perfil cromatográfico del aceite se calculó con el método de normalización de áreas. La cuantificación de los compuestos específicos se hizo comparando su valor con el del compuesto estándar correspondiente.

### Cultivo y mantenimiento del microorganismo evaluado

Se evaluó la cepa *C. acnes* ATCC 6919 depositada en la *American Type Culture Collection* (ATCC) como *Corynebacterium acnes* (Gilchrist) Ebersony, más recientemente denominada *Cutibacterium acnes Scholz and Kilian* ATCC 6919™ ^18^^,^^19^. Se utilizó un inóculo preservado de un primer pase a -70 °C, el cual se reactivó en el medio de cultivo sólido agar Schaedler; a continuación, se hizo un nuevo pase en el mismo medio para el mantenimiento de la cepa.

Los cultivos se incubaron en condición de anaerobiosis a una temperatura de 37 °C durante 96 horas. A partir de este cultivo, se preparó un inóculo diluido en solución salina estéril, ajustado a un patrón 0,5 de McFarland calculado por nefelometría, según lo descrito en los protocolos del *Clinical and Laboratory Standards Institute* (CLSI) ^20^.

### Evaluación de la actividad antimicrobiana contra Cutibacterium acnes

La actividad antimicrobiana del aceite del árbol de té se evaluó mediante un ensayo de dilución en agar, tal como lo describe la guía M11-A8 del CLSI ^20^. Para este fin, se preparó agar Mueller-Hinton con suplemento al 5 % de sangre de carnero, 5 ug/ml de hemina y 1 ug/ml de vitamina k. En la evaluación inicial del aceite en concentración al 2 % v/v, se diluyó el producto puro en el medio de cultivo en una proporción adecuada para obtener la concentración correcta, y el medio de cultivo con el aceite embebido se sirvió en placas de Petri (placas de dilución). Para la determinación de la CIM, se hicieron diluciones adecuadas del aceite del árbol de té en aceite mineral y se obtuvieron concentraciones sucesivas del 2, 1, 0,5, 0,25, 0,125 y 0,0625 % v/v que, posteriormente, se añadieron al medio de cultivo y se sirvieron en placas de Petri ^20^.

En todas las pruebas se incluyó un control de crecimiento en el medio de cultivo (inóculo microbiano), un control negativo (3 µg de ampicilina) y un control de crecimiento en el vehículo de dilución (aceite mineral), este último con el fin de descartar la inhibición del crecimiento por acción del aceite mineral. El microorganismo se sembró tomando 10 µl inóculo ajustado a un patrón de 0,5 de McFarland, depositándolo sobre el agar y dejándolo reposar en el medio de cultivo hasta que la gota de inóculo se secara.

Los cultivos se incubaron en condición de anaerobiosis a una temperatura de 37 °C durante 96 horas, al cabo de las cuales se determinó la presencia o ausencia de crecimiento. La CIM se determinó como la menor concentración del producto que inhibiera el crecimiento visible del microorganismo ^21^.

Las pruebas de actividad antimicrobiana en concentración al 2 % v/v y de la CIM, se realizaron con cada lote por triplicado y, en todos los casos, se trabajaron los respectivos controles.

## Resultados

Las características descritas para los tres lotes de aceite del árbol de té: color, densidad e índice de refracción, cumplieron con lo establecido por la norma ISO 4730 ^4^. En cuanto a los componentes de los tres lotes de aceite del árbol de té y la cantidad de cada uno, se encontró que el terpinen-4-ol fue el principal componente, con una concentración entre 40,7 y 43,9 %. El segundo componente más abundante fue el y-terpineno, con valores entre 20,7 y 22,6 %, seguido del a-terpineno, con una concentración entre 9,5 y 10,5 %. También, el componente p-cineole representó una cantidad mínima de 2,3 a 3,0 %. Los demás componentes de los aceites se encontraron en mínimas cantidades y todos cumplieron con el rango establecido en la norma ISO 4730 ^4^^,^^5^. La composición y las características de los lotes evaluados se presentan en el cuadro 3.

**Cuadro 3:** Características y composición de los lotes de aceite del árbol de té evaluados

Característica	Valores estándar*	Lote 1 1506107	Lote 2 1510251	Lote 3 1508336
Fecha de análisis	-	08/02/2017	18/09/2017	24/06/2017
Color	Incoloro/amarillo pálido	Correcto	Correcto	Correcto
Densidad (g/ml)	0,885-0,906	0,895	0,896	0,896
Índice de refracción	1,4750-1,4820	1,4779	1,4771	1,4784
Composición (%)				
Terpinen-4-ol	39,0-45,0	43,9	40,7	42,9
α-pineno	1,0-6,0	2,4	2,4	2,3
Sabineno	0,0-3,5	0,6	0,1	0,3
α -terpineno	0,5-13,0	10,5	9,5	10,3
Limoneno	0,5-1,4	0,7	1,0	0,7
p-cimeno	0,5-4,0	2,0	2,0	3,7
p-cineol	0,0-5,0	2,7	3,0	2,3
y-terpineno	10,0-28-0	22,6	20,7	22,3
α -terpinoleno	1,5-5,0	3,2	3,5	3,4
a-terpineol	1,5-8,0	2,8	3,0	2,6
Aromadendreno	0,0-7,0	0,8	1,3	1,1
d-cadineno	0,0-8,0	1,0	1,0	1,3
Globulol	0,0-3,0	1,0	0,4	0,1
Viridiflorol	0,0-1,5	0,0	0,3	0,1

* 5, 6

Para la evaluación de la actividad antimicrobiana del aceite del árbol de té, inicialmente se evaluó en una concentración de 2 % v/v contra la cepa de *C. acnes* ATCC 6919. En la prueba se observó un efecto inhibitorio del crecimiento del microorganismo en todos los platos de los diferentes lotes del aceite a esta concentración. Además, hubo crecimiento de *C. acnes* en los platos de control de crecimiento en el medio de cultivo y del vehículo de dilución (no se presentan los datos). Dados estos resultados, se evaluó la CIM teniendo en cuenta un valor máximo de la concentración de 2 % v/v.

**Figura 1:**
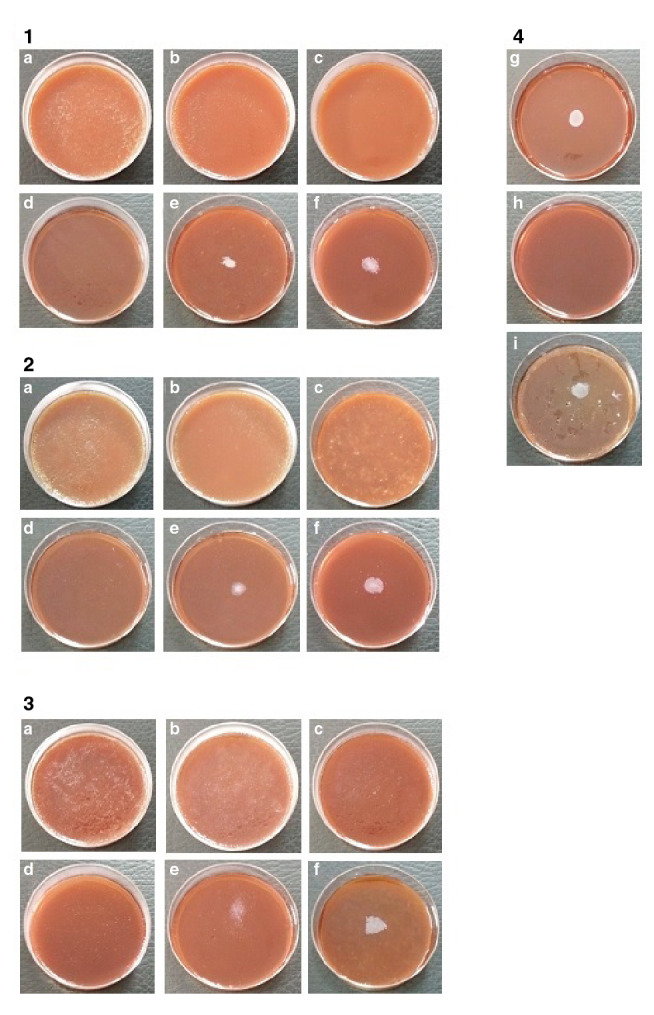
Concentración inhibitoria mínima (CIM) para cada lote de aceite del árbol de té contra *Cutibacterium acnes* ATCC 6919 después de tres días de incubación. 1) Aceite del árbol de té, lote 1 (1506107). 2) Aceite del árbol de té, lote 2 (1510251). 3) Aceite del árbol de té, lote 2 (1508336): (a) 2 % v/v, (b) 1 % v/v, (c) 0,5 % v/v, (d) 0,25 % v/v, (e) 0,125 % v/v, (f) 0,0625 % v/v. 4) Controles de calidad: control de crecimiento en el medio de cultivo (g), control negativo (h), control de crecimiento en el vehículo de dilución (i)

El resultado de la CIM contra *C. acnes* se presenta en la figura 1. A los tres días de incubación, y en los tres lotes evaluados, se presentó crecimiento en los platos con aceite del árbol de té al 0,0625 y 0,125 % v/v, pero no hubo presencia del microorganismo en los platos con el aceite al 0,25, 0,5, 1 y 2 % v/v en ninguna de las tres repeticiones. Por consiguiente, la CIM contra *C. acnes* fue de 0,25 % v/v, sin diferencias en sus valores entre los tres lotes.

Al igual que en las pruebas con concentración al 2 % v/v, los controles utilizados tuvieron el comportamiento esperado: ausencia de crecimiento de *C. acnes* en el control negativo y crecimiento homogéneo del control de crecimiento en el medio de cultivo y en el vehículo de dilución (figura 1).

## Discusión

La norma ISO 4730 establece los criterios físicos y químicos que debe cumplir un aceite esencial del árbol de té que tenga el quimiotipo idóneo ^5^. En la literatura especializada se describen varios quimiotipos de este aceite esencial, pero el que contiene entre 30 y 40 % del componente terpinen-4-ol es el que comúnmente se acepta para la comercialización. Los análisis evidenciaron que los tres lotes de aceite estudiados cumplían con dicha característica y que el porcentaje de este componente, principal responsable de la actividad antimicrobiana ^2^^,^^6^, era ligeramente mayor (40,7, 42,9 y 43,9 %).

En los últimos años, se ha incrementado el uso del aceite del árbol de té como alternativa terapéutica en diversas enfermedades, especialmente las de la piel como el acné vulgar, gracias a su efecto antimicrobiano contra *C. acnes*^1^. Los resultados del presente estudio indican que el aceite del árbol de té tiene actividad antimicrobiana contra *C. acnes,* con un efecto dependiente de la concentración y una CIM de 0,25 % v/v, a partir de la cual se inhibió su crecimiento eficazmente. Este hallazgo es similar a los de estudios anteriores sobre la CIM del aceite del árbol de té contra *C. acnes* que utilizaron el mismo método y obtuvieron un rango entre 0,05 y 2,0 % v/v ^14^^,^^22^^-^^24^.

En este sentido, en el estudio de Luangnarumitchai, *et al.,* sobre la actividad antibacteriana de 22 aceites esenciales, incluido el del árbol de té, se demostró una CIM del 1 % v/v para cinco cepas diferentes de *C. acnes*^23^. Asimismo, Griffin, *et al.,* estandarizaron un método de dilución en agar para la prueba de sensibilidad de dos aceites de *M. alternifolia* con diferentes concentraciones de terpinen-4-ol (37 y 45 % v/v) en una amplia gama de bacterias y hongos. Los autores reportaron una CIM para *C. acnes* de 0,5 % v/v para el aceite con 37 % del componente activo y entre 0,4 y 0,5 % v/v para el aceite con un 45 % de terpinen-4-ol ^24^. Por su parte Lee, *et al.,* exploraron la correlación de los componentes del aceite del árbol de té con su actividad antibacteriana contra *C. acnes* y S. *aureus,* y establecieron una CIM de 0,625 y 1,25 % v/v, respectivamente ^3^.

En el presente estudio, se confirmó que el aceite del árbol de té evaluado presentaba una actividad antimicrobiana eficiente contra *C. acnes* y que el valor de la CIM era cercano al menor valor de los rangos reportados en la literatura, lo que respondería al contenido ligeramente mayor de terpinen-4-ol, dado que la actividad antimicrobiana del aceite se atribuye principalmente a este componente ^2^.

Esta hipótesis se ve respaldada por un estudio que exploró las correlaciones de los componentes del aceite del árbol de té con su actividad contra el acné vulgar y la irritación de la piel. El aceite se probó contra S. *aureus* y *C. acnes,* y se comprobó que el terpinen-4-ol y el α-terpineol presentaban una acentuada actividad antimicrobiana contra *C. acnes.* En cuanto a p-cineole, este se encontró en una cantidad moderada en los tres lotes de aceite evaluados, lo cual resulta importante si se considera que los que se comercializan deben tener una cantidad menor del 15 % de dicho componente debido a que es irritante para la piel ^3^.

Todos los estudios citados se llevaron a cabo utilizando el método de dilución en agar y, a pesar de que este se desarrolló para el trabajo con agentes antimicrobianos solubles en agua, ha sido el método de elección en muchos de los estudios de comparación de la actividad antimicrobiana de los aceites esenciales ^14^^,^^22^^,^^23^.

En las investigaciones mencionadas y en el presente estudio, el desempeño de la prueba fue satisfactorio y el vehículo de dilución y los demás elementos del medio de cultivo no afectaron la acción antimicrobiana del aceite del árbol de té, lo que resultó en un crecimiento satisfactorio de *C. acnes* en las placas de Petri (control de crecimiento en el medio de cultivo y control de crecimiento en el vehículo de dilución) y en ausencia de crecimiento en el control negativo, lo que garantiza la calidad, la funcionalidad y la confiabilidad del método para evaluar la actividad antimicrobiana.

Es importante mencionar que el tratamiento tópico del acné con aceite del árbol de té puede causar irritación de la piel y las mucosas y que las reacciones irritantes o alérgicas dependen de la concentración utilizada, según se ha reportado en diversos estudios ^25^. En uno realizado *in vivo,* se reporta que la irritación de la piel se redujo significativamente cuando las concentraciones del aceite fueron de 2,5 % v/v o menos ^3^.

Lo anterior deja claro que, para el cuidado de la piel, es importante tener un producto seguro y eficaz al mismo tiempo. La CIM encontrada en los lotes de aceite evaluados en este estudio permite utilizarlos de manera más eficiente por la levemente elevada concentración de sus componentes y la consecuente disminución del riego de reacciones alérgicas o irritación. Sin embargo, es necesario hacer nuevos estudios con estos productos en las concentraciones de CIM halladas contra otras cepas de *C. acnes* y aislamientos provenientes de pacientes con lesiones de acné, para confirmar su actividad. Asimismo, deben hacerse estudios clínicos para comprobar la reducción de posibles efectos adversos, como la irritación, en las concentraciones formuladas.

Los resultados aquí obtenidos indican que cualquiera de los tres lotes de aceite del árbol de té evaluados inhibiría eficazmente el crecimiento de *C. acnes* y que existe homogeneidad en su actividad antimicrobiana en concentraciones de 2 % v/v o menos y con una CIM de 0,25 % v/v, valor que se encuentra en el rango de 0,05 a 2 % v/v descrito en otros estudios.

En conclusión, el aceite del árbol de té es un producto que inhibe eficazmente el crecimiento de *C. acnes* y tiene un importante potencial para su uso solo o incorporado en cosméticos y productos farmacéuticos.
